# Multicentre phase II trial of capecitabine plus oxaliplatin (XELOX) in patients with advanced hepatocellular carcinoma: FFCD 03-03 trial

**DOI:** 10.1038/sj.bjc.6603956

**Published:** 2007-09-18

**Authors:** V Boige, J-L Raoul, J-P Pignon, O Bouché, J-F Blanc, L Dahan, J-L Jouve, N Dupouy, M Ducreux

**Affiliations:** 1 Gastrointestinal Oncology Unit, Institut Gustave Roussy, Villejuif, France; 2 Gastrointestinal Oncology Unit, Centre Eugene Marquis, Rennes, France; 3 Biostatistics Unit, Institut Gustave Roussy, Villejuif, France; 4 Gastrointestinal Unit, University Hospital, Reims, France; 5 Hepato-Gastroenterology Unit, University Hospital, Bordeaux, France; 6 Digestive Oncology Unit, la Timone Hospital and University of the Mediterranean, Marseille, France; 7 Gastrointestinal Unit University Hospital, Dijon, France

**Keywords:** hepatocellular carcinoma, chemotherapy, phase II clinical trial, capecitabine, oxaliplatin, XELOX

## Abstract

Evaluation of new drug combinations is needed to improve patients' prognosis in advanced hepatocellular carcinoma (HCC). The purpose of this study was to evaluate the safety and efficacy of the capecitabine–oxaliplatine combination (XELOX) in HCC patients. First-line chemotherapy with XELOX regimen consisting of a 3-week cycle of intravenous oxaliplatin (130 mg m^−2^) on Day 1, and oral capecitabine twice daily from Days 1–14 (1000 mg m^−2^) was administered in patients with measurable, unresectable HCC. Fifty patients (male, 88%; median age, 68 years) received a total of 295 cycles (median, 6) of treatment. Disease control (three partial responses, 29 stable diseases) rate was 72% (95% CI 57–83%). Median overall and median progression-free (PFS) survival was 9.3 months and 4.1 months, respectively. Progression-free survival rates at 6 and 12 months were 38% (95% CI 26–52%) and 14% (95% CI 7–26%), respectively. Main grade 3–4 drug-related toxicities included diarrhoea (16%), elevation of aminotransferases and/or bilirubin (16%), thrombocytopenia (12%), and neurotoxicity (6%). Capecitabine plus oxaliplatin regimen showed modest anti-tumour activity with tolerable toxicities in patients with advanced HCC. However, the manageable toxicity profile and the encouraging disease control rate deserve further attention for this convenient, outpatient-based chemotherapy regimen.

Worldwide, hepatocellular carcinoma (HCC) is the fifth most common cancer and the third most common cause of cancer-related death (Parkin *et al*, 2001). It is seen primarily in the setting of chronic liver disease or cirrhosis. As a result of the tumour extent and/or underlying decompensated cirrhosis at time of diagnosis, only a few patients are eligible for radical treatments with curative intent (ie, surgical resection, liver transplantation, percutaneous ablation). Chemoembolisation, which has been shown to improve survival in selected patients (Bruix *et al*, 2004), is not feasible in case of portal vein thrombosis or illogical in case of extrahepatic spread. Therefore, a substantial proportion of patients with advanced HCC are eligible for palliative systemic therapy. However, no chemotherapeutic agent including doxorubicin alone or in combination has produced a substantial improvement in patient survival rates as a result of poor response rate and increased toxicity (Lai *et al*, 1988; Burroughs *et al*, 2004; Yeo *et al*, 2005).

Unlike other platinum salts, oxaliplatin has consistently shown preclinical and clinical anti-tumour activity against gastrointestinal cancers without nephrotoxicity. In colon cancer it is well known that the combination of 5-fluorouracil (5-FU), either as a short or continuous infusion combined with oxaliplatin gives improved overall response rate compared to 5-FU alone (de Gramont *et al*, 2000). Sensitive peripheral neuropathy is the most frequent limiting toxicity with oxaliplatin, but this is reversible with treatment discontinuation (Brienza *et al*, 1995). A pilot study conducted by Bearz *et al* (2001) and updated by Frustaci *et al* (2003) in 31 patients suffering from inoperable or metastatic HCC showed the feasibility and demonstrated some efficacy of 5-FU/oxaliplatin (FOLFOX) combination with an overall response rate of 29%.

Capecitabine is a rationally designed, orally administered, tumour-selective fluoropyrimidine that mimics continuous infusion of 5-FU. Capecitabine is converted to 5-FU preferentially in tumour tissue by the enzyme thymidine phosphorylase (Miwa *et al*, 1998; Schuller *et al*, 2000) and has been approved by the Food and Drug Administration and the European Agency for the Evaluation of Medicinal Products (EMEA) to treat patients with colorectal cancer (CRC). The efficacy and toxicity of capecitabine in 37 patients with unresected HCC was analysed in one retrospective study reported by Patt *et al* (2004); capecitabine was found to be safe in patients with cirrhosis and provided an 11% response rate including one radiologically confirmed complete response (Cassidy *et al*, 2004).

The rationale for developing the capecitabine–oxaliplatin combination in HCC was based on (1) the synergy of these two drugs (Cassidy *et al*, 2004); (2) the lack of renal toxicity of oxaliplatin in cirrhotic patients (low risk of oedema and ascites due to non-required hyperhydration) (Brienza *et al*, 1995); (3) the clinical activity and favourable toxicity profile of capecitabine alone and in combination with oxaliplatin in advanced or metastatic CRC (Van Cutsem *et al*, 2001; Borner *et al*, 2002; Cassidy *et al*, 2004); and (4) no dose adjustment required for capecitabine and oxaliplatin in hepatic dysfunction (Brienza *et al*, 1995; Twelves *et al*, 1999). This French, multicentre, open-label, phase II study aimed to assess efficacy and safety of first-line capecitabine plus oxaliplatin (XELOX) regimen in patients with unresectable HCC without decompensated cirrhosis.

## PATIENTS AND METHODS

### Patients

Patients who were not suitable for surgical resection, liver transplantation, or local ablation techniques, with either histologically proven HCC, or combination of liver cirrhosis, radiologically documented hypervascular liver tumour and alphafetoprotein (AFP) level>400 ng ml^−1^, were eligible to this open-label, phase II study. Other eligibility criteria were measurable tumour mass ⩾2 cm in diameter; Cancer of the Liver Italian Program score (CLIP) (cancer of the liver program investigators, 1998) <4; age>18 years; World Health Organization (WHO) performance status (PS) 0–2; Child–Pugh score of A or B; life expectancy >12 weeks; adequate hepatic, renal and bone marrow function (serum bilirubin ⩽1.5 the upper limit of normal (ULN), creatinine ⩽1.5 ULN, neutrophils>1.5 g l^−1^, platelets>75 g l^−1^). Main exclusion criteria were Child–Pugh score of C, previous systemic chemotherapy, chemoembolisation or embolisation, or radiotherapy; concomitant anti-tumour therapy including tamoxifen, interferon and somatostatin analogues, central nervous system metastases, severe cardiac and/or respiratory failure, concurrent malignancy, and baseline sensitive peripheral neuropathy; and pregnant or lactating females. Patients provided written informed consent.

### Study design

This was a multicentre, open-label phase II study conducted at six centres in France. The study protocol was approved by an independent Ethics Committee (Comité Consultatif de Protection des Personnes dans la Recherche Biomédicale, C.C.P.P.R.B. at Bicêtre Hospital, Paris, France) and carried out according to International Conference on Harmonisation/WHO Good Clinical Practice standards Guidelines and declaration of Helsinki.

### Treatment protocol

Capecitabine plus oxaliplatin regimen was administered as a 3-week cycle. In each cycle, oxaliplatin was administered at a total dose of 130 mg m^−2^ as a 2-h i.v. infusion on Day 1, and capecitabine 1000 mg m^−2^ was taken orally twice daily (total daily dose 2000 mg m^−2^) on Days 1–14.

Complete blood cell and platelet counts were performed weekly, and physical examination, biology (serum AFP, transaminases, alkaline phosphatases, bilirubin, lactate dehydrogenase, *γ*-glutamyl transferase, albumin, prothrombin time (PT), and creatinine), and safety assessments were performed before each cycle of chemotherapy. Analysis of AFP level and tumour assessment (computed tomography (CT) scan or magnetic resonance imaging) were undertaken every three cycles. Objective response (OR) was confirmed by a second evaluation 4 weeks later. Objective and discordant responses were reviewed by an independent radiologist. Study treatment was continued until either disease progression according to Response Evaluation Criteria in Solid Tumours (RECIST) (Therasse *et al*, 2000); unacceptable toxicity; or patient's refusal. After cessation of study treatment, second-line therapy of HCC was at the investigator's discretion.

### Toxicity assessment and dose modification

Safety was assessed according to the National Cancer Institute – Common Terminology Criteria for Adverse Events version 2.0, but any fatal or non-fatal decompensation of cirrhosis was considered separately. The oxaliplatin-specific scale was used to assess oxaliplatin neurotoxicity (Caussanel *et al*, 1990). In cases of non-neurological grade 3–4 toxicity, oxaliplatin and capecitabine were reintroduced at the following cycle only after recovery to grade 0–1 toxicity with a 20% dose reduction after the first occurrence, a 40% dose reduction after the second episode and treatment cessation after the third episode.

In cases of grade 2 sensory neuropathy (defined as dysaesthesia/paraesthesia persisting over two cycles, without dysfunction), a 20% dose reduction was applied to oxaliplatin in the subsequent cycle, whereas in case of grade 3 neuropathy (defined as permanent functioning discomfort) oxaliplatin was stopped and capecitabine was administered alone.

### Statistical analysis

The main objective of the study was to assess OR rate according to RECIST classification of the XELOX combination. Secondary objectives were to assess response and/or stabilisation durations, progression-free survival (PFS), overall survival (OS), and safety profile of the combination. A one-step Fleming design with a 5% type I error (one-sided) 95% power, a 10% OR rate as null hypothesis, and a 30% as alternative hypothesis was used (Fleming, 1982; Machin *et al*, 1997). The planned accrual was for 40 patients. Because of frequent early progression or decompensated liver disease in patients with HCC and cirrhosis, the number of patients included was increased to 50 patients. Disease progression was defined as the time from the start of therapy until tumour progression or death whatever its cause, and OS from the start of therapy to the date of the death or last follow-up. Time-to-event parameters were analysed using Kaplan–Meier product limit estimates, and between–group comparisons were performed using the Log-rank test. Cox regression model was used for multidimensional analysis. All reported *P*-value were two-sided.

## RESULTS

### Patient characteristics

Fifty patients (44 men, 6 women), median age 68 years (range: 24–82 years), were included in the trial between December 2003 and September 2004. Patients' baseline characteristics are shown in Table 1. Thirty-three patients (66%) had a history of alcohol abuse either alone (28 patients) or combined with either haemochromatosis (two patients), hepatitis B virus (HBV) (two patients) or hepatitis C virus (HCV) (one patient). Aetiology was viral in eight other patients (16%): HCV for five patients and HBV for three patients. Cirrhosis was present in 36 patients (72%) and 9 patients (18%) had normal serum AFP at baseline (missing in one patient).

### Response to treatment and survival

**Figure 1:**
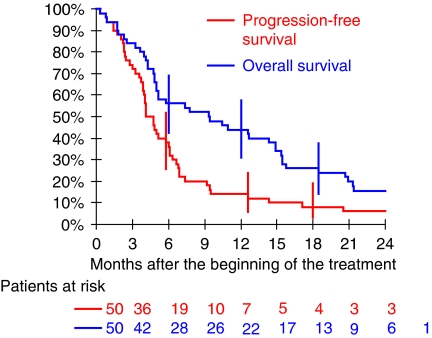
Kaplan–Meier estimation of progression-free survival and overall survival (*N*=50).

A total of 295 cycles of chemotherapy were administered. The median number of cycles per patient was 6 (range: 1–21 cycles); 12 patients received less than three cycles. In total, the median (range) dose received per patient was 10 304 mg m^−2^ (1856–37 941) for capecitabine and 681 mg m^−2^ (100–2507) for oxaliplatin. The median (range) dose per treatment day was 1952 mg m^−2^ (930–2128) for capecitabine and 128 mg m^−2^ (0–137) for oxaliplatin (minimum of 78 mg m^−2^ if administered). Forty-one (82%) patients were evaluable for tumour response. Among the nine remaining patients, four patients (8%) received only one cycle of chemotherapy and five patients (10%) two cycles. The cause of death of these patients was liver cancer for seven, liver disease for one, and toxicity for the other. The best tumour response was partial response (PR) in 3 patients (7%), stable disease (SD) in 33 patients (81%), and disease progression in 5 patients (12%). Partial response duration in the three patients was 1.1, 5.0, and 7.3 months respectively, whereas duration of SD ranged from 2.2 to 20.5 months (median: 5.4 months). In the intention-to-treat group (*N*=50), the tumour control rate (PR and SD) was 72% (95% confidence interval (CI) 57–83%). The tumour control rate was 77% (95% CI 61–88%) in the 43 patients with Child–Pugh A score cirrhosis, including the three patients with PR. Among the 36 patients with at least two assessments of serum AFP, one complete and four partial biological responses (defined by normalisation and decrease level more than 50%, respectively) were observed. All patients ended their XELOX treatment, the last one ending on January 2006. The cutoff date for analysis was 1 July 2006. Median patient follow-up using inverted Kaplan–Meier method was 26 months and ranged from 21 to 29 months in patients alive. Six patients were alive at the cutoff date (all with disease progression). Hepatocellular carcinoma was the cause of death in 37 (84%) out of the 44 patients with progression (combined with cirrhosis decompensation in four patients (9%)), cirrhosis decompensation alone in four patients (9%), toxic death in two patients (5%), and cardiac failure in one patient (2%). Median PFS and OS were 4.1 and 9.3 months, respectively. Estimated PFS was 38% (95% CI: 26–52%) at 6 months and 14% (7–26%) at 12 months (Figure 1), whereas OS was 56% (42–69%) at 6 months, 44% (31–58%) at 12 months, 26% (16–40%) at 18 months, and 15% (8–28%) at 24 months (Figure 1).

**Figure 2:**
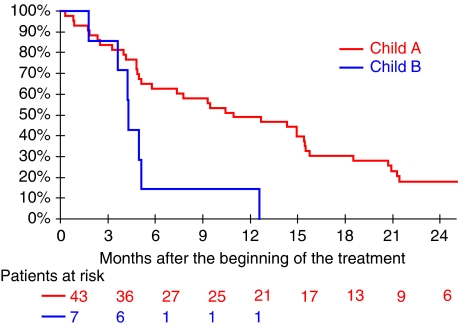
Kaplan–Meier estimation of overall survival by Child–Pugh score group (*N*=50).

By univariate analysis in the overall population, OS was significantly longer in patients with a Child–Pugh score of A compared with score B patients (*P*=0.0076), with a median OS of 10.4 *vs* 4.3 months (Figure 2), and there was a trend towards longer OS (*P*=0.056) in patients whose baseline PS was equal to zero compared with other patients (PS⩾1). There were no significant effects of age, CLIP score, and presence of clinical cirrhosis or alcohol abuse on OS (data not shown). Using multivariate analysis (Cox model), hazard ratio of death was 3.01 (95% CI: 1.25–7.26) in patients with a Child–Pugh score of B compared with those patients with a score of A (*P*=0.014) (Table 2).

### Toxicity

All 50 patients but one who received only one cycle were evaluable for toxicity. In total, 30 patients (61%) experienced at least one grade 3 or 4 treatment-related toxicity (Table 3). The most frequent grade 3–4 nonhaematologic toxicities were diarrhoea, elevation of aminotransferases, and/or bilirubin, and fatigue. Hand-foot syndrome was severe in only two patients. Grade 2 and 3 peripheral neuropathy was observed in 11 (22%) and 3 (6%) patients, respectively. Only two patients had a severe neutropenia. Six patients (12%) had grade 3–4 thrombocytopenia. The reason for treatment withdrawal was disease progression in 30 patients (60%), decompensated cirrhosis in 7 patients (14%), cardiac failure in 1 patient (2%), treatment-related toxicity in 5 patients (10%), patient's refusal in 4 patients (8%), and other reason not otherwise specified in 3 patients (6%). Two treatment-related deaths were observed including one patient with myocardial infarction concomitant with neutropenic infection and grade 3 diarrhoea after the first course of treatment, and one patient with neutropenic pneumopathy after the third course of treatment.

## DISCUSSION

There is no systemic chemotherapy that can be considered as a standard for advanced HCC as no drug or combination has been convincingly shown to improve survival over best supportive care (Lai *et al*, 1988; Burroughs *et al*, 2004). In addition to intrinsic resistance, underlying liver cirrhosis most often precludes the use of several cytotoxic agents, namely cytotoxic agents metabolised by the liver and excreted into the bile. The lack of renal toxicity of oxaliplatin (Brienza *et al*, 1995), the low incidence of myelosuppression observed with capecitabine (Van Cutsem *et al*, 2001), the synergistic anti-tumour activity of capecitabine and oxaliplatin combination in advanced gastrointestinal cancers (Cassidy *et al*, 2004; Park *et al*, 2006b), and the absence of dose adjustment required for both agents in case of hepatic dysfunction (Brienza *et al*, 1995; Twelves *et al*, 1999) make the XELOX regimen attractive in cirrhotic patients with HCC.

In the current study, XELOX regimen showed modest activity in patients with advanced HCC using the RECIST criteria. Thus, although it is difficult to compare the efficacy results from one to another study because of heterogeneous tumour response criteria and patient selection, our study did not confirm previous results of clinical activity reported with capecitabine alone or FOLFOX regimen (Frustaci *et al*, 2003; Patt *et al*, 2004). Higher tumour response rates of 15–25% were previously obtained with doxorubicin and cisplatin combinations with either capecitabine or UFT. However, this did not seem to affect significantly PFS and OS found to be less than 4 months and 8 months, respectively (Kim *et al*, 2006; Park *et al*, 2006a). A recent randomised phase III study comparing single agent doxorubicin *vs* PIAF regimen (cisplatin/interferon a-2b/doxorubicin/fluorouracil) did not show any significant difference in OS between the two arms despite borderline statistical significance in favour of PIAF (6.8 and 8.7 months for doxorubicin and PIAF arms, respectively) (Yeo *et al*, 2005). Therefore, no drug or combination has been shown to be better than single agent doxorubicin, which does not convincingly improve survival over supportive care. Thus, one can argue that there is no standard chemotherapy and that supportive care has to be the control group of any randomised trial (Burroughs *et al*, 2004).

With a disease control rate of 72%, a median survival of 9.3 months, and a one-year survival rate of 44%, the XELOX regimen compares favourably with other systemic therapies for HCC. The 6 months PFS rate was 38%, suggesting that XELOX regimen was capable of achieving durable stabilisation of advanced-stage HCC. As assessment of response to treatment based on conventional criteria that relies mainly on radiological evaluation may not be reliable in advanced HCC (Yeo *et al*, 2005), PFS may be a more appropriate and informative primary end point of further phase II studies. This is all the more true and already the case for phase II studies assessing the efficacy of new targeted therapies that may be cytostatic instead of cytotoxic (Philip *et al*, 2005; Abou-Alfa *et al*, 2006). As an example, recent phase III results of sorafenib, a multikinase inhibitor targeting Raf kinase, vascular endothelial growth factor receptor, and platelet-derived growth factor receptor, have demonstrated a statistically significant improvement in OS and time to progression (TTP) in a comparable patient population with advanced HCC, despite a low response rate according to the RECIST criteria observed in a previous phase II study (Abou-Alfa *et al*, 2006; Llovet *et al*, 2007): median OS was 10.7 months in the sorafenib group as compared to 7.9 months in the placebo group (HR=0.69, *P*=0.0006), and median TTP was 5.5 *vs* 2.8 months, respectively (HR=0.58, *P*=0.000007).

The toxicity profile was acceptable in our trial including patients with HCC and underlying cirrhosis. Haematologic toxicity was mild compared with that observed with anthracyclin-based polychemotherapy regimens. Grade 3–4 thrombocytopenia and neutropenia was observed in 12 and 4% of the population, respectively. Although no direct comparison with our previous phase II with gemcitabine/oxaliplatin regimen, the use of capecitabine instead of gemcitabine in combination to oxaliplatin resulted in lower haematological toxicity (Louafi *et al*, 2007). Of note, the threshold value of platelet count as an inclusion criteria was decrease to 75 g l^−1^ because of frequent baseline portal hypertension associated thrombocytopenia under 100 g l^−1^. Despite a potential risk of bleeding from oesophageal varices, yet no bleeding episodes occurred, possibly owing to close monitoring and dose adjustment. We also observed liver dysfunction as measured by an increase in hepatic transaminase and hyperbilirubinemia. However, severe liver test abnormalities may be due, in part, to coexisting chronic liver disease since oxaliplatin and capecitabine were not found to be hepatotoxic in patients with CRC except benign and unexplained isolated hyperbilirubinemia occasionally observed under capecitabine treatment (Van Cutsem *et al*, 2001). Otherwise, no HBV reactivation was observed despite no systematic lamivudin prophylaxis in HBsAg-positive patients.

We also conducted exploratory analysis of prognostic factors. Only the Child–Pugh score was consistently associated withOS. These findings are consistent with those of previous reports in which low bilirubin, high albumin level, and absence of ascites were associated with improved survival of HCC patients after chemotherapy, as those factors are included in the Child–Pugh score (Okada 1998; Leung *et al*, 2002; Yeo *et al*, 2005). Interestingly, albumin level has also been shown to be a prognostic factor of treatment outcome in HCC patients receiving doxorubicin-based regimen (Yeo *et al*, 2005). Stratification of patients according to specific prognostic scores used in HCC and cirrhosis should be included in future trials designed to better identify patients who may benefit from systemic chemotherapy.

## CONCLUSION

Despite a disease control rate of 72% and a favourable toxicity profile, XELOX regimen had a modest anti-tumour activity for patients with advanced HCC and cirrhosis. Future phase II and III trials should assess the efficacy of new promising treatment approaches such as molecularly targeted therapies alone or in combination with cytotoxic agents using appropriate primary end point, and also define predictive biomarkers according to the drug's mechanism of action and the biologic behaviour of the tumour.

## Figures and Tables

**Table 1:** Patients' baseline characteristics

**Characteristics**	***N*=50**
Median age, years (range)	68 (24–82)
	
*Gender*	
Male, *n* (%)	44 (88)
Female, *n* (%)	6 (12)
	
*World Health Organization PS, n* (%)	
0	22 (44)
1	25 (50)
2	3 (6)
	
*Cancer Liver Italian Program score, n* (%)	
0	1 (2)
1	18 (36)
2	19 (38)
3	12 (24)
	
*Child–Pugh score, n* (%)	
A	43 (86)
B	7 (14)
Coexisting cirrhosis, *n* (%)	36 (72)
	
*Aetiology of liver disease, n* (%)	
Alcohol^a^	33 (66)
Hepatitis C	5 (10)
Hepatitis B	3 (6)
Hemochromatosis	4 (8)
Other^b^	5 (10)
Median AFP^c^, ng ml^−1^ (range)	159 (11–487 221)

AFP=alphafetoprotein.

a Alcohol abuse: alone in 28 patients (56%), with either haemochromatosis (two patients, 4%) or hepatitis B virus (two patients, 4%) or hepatitis C virus (one patient, 2%).

b Normal liver in two patients (4%); not otherwise specified, unknown origin and degenerated adenomatosis in one patient (2%), each.

c In the 40 patients with abnormal AFP level at baseline.

**Table 2:** Univariate and multivariate analyses of overall survival (*N*=50)

		**Univariate analysis**		**Multivariate analysis**	
	***n***	**12-month OS (95% CI)**	***P*-value^a^**	**HR (95% CI)**	***P*-value^b^**
*Child–Pugh score*					
A	43	49% (35–63%)	**0.0076**	1	**0.0140**
B	7	14% (3–51%)		3.01 (1.25–7.26)	
					
*PS (WHO)*					
0	22	59% (39–77%)	0.056	1	0.070
1–2	28	32% (18–51%)		1.76 (0.95–3.26)	

OS=overall survival; CI, confidence interval; HR=hazard ratio; PS=performance status.

a Log-rank test.

b Cox model.

Bold values signifies <0.05.

**Table 3:** Treatment-related toxicities in 49 patients

	**Grade 1–2**	**Grade 3–4**
	**No. of patients (%)**	**No. of patients (%)**
Thrombocytopenia	17 (35%)	6 (12%)
Transaminases or bilirubin	31 (63%)	8 (16%)
Diarrhoea	18 (37%)	8 (16%)
Anaemia	31 (63%)	6 (12%)
Neurotoxicity	37 (76%)	3 (6%)^a^
Nausea/vomiting	26 (53%)	2 (4%)
Neutropenia	14 (29%)	2 (4%)
Hand foot syndrome	17 (35%)	2 (4%)
Mucocitis	7 (14%)	1 (2%)
Other	29 (59%)	6 (12%)^b^
		
*Toxic deaths*		
Myocardial infarction in neutropenia	1 (6%)	
Aplastic pneumopathy	1 (6%)	
		

a At cycles 8, 9, and 11, respectively.

b Four fatigue, one fatigue and lombalgia, one fatigue and anorexia, one fatigue and hypokalemia, one liver pain, one hypercreatininemia, one leucopenia, one constipation, and one diabetes decompensation.
